# Event-Based Sensing and Signal Processing in the Visual, Auditory, and Olfactory Domain: A Review

**DOI:** 10.3389/fncir.2021.610446

**Published:** 2021-05-31

**Authors:** Mohammad-Hassan Tayarani-Najaran, Michael Schmuker

**Affiliations:** School of Physics, Engineering and Computer Science, University of Hertfordshire, Hatfield, United Kingdom

**Keywords:** event based signal processing, signal processing, artificial retina, artificial olfactory, artificial cochlea, machine leading

## Abstract

The nervous systems converts the physical quantities sensed by its primary receptors into trains of events that are then processed in the brain. The unmatched efficiency in information processing has long inspired engineers to seek brain-like approaches to sensing and signal processing. The key principle pursued in neuromorphic sensing is to shed the traditional approach of periodic sampling in favor of an event-driven scheme that mimicks sampling as it occurs in the nervous system, where events are preferably emitted upon the change of the sensed stimulus. In this paper we highlight the advantages and challenges of event-based sensing and signal processing in the visual, auditory and olfactory domains. We also provide a survey of the literature covering neuromorphic sensing and signal processing in all three modalities. Our aim is to facilitate research in event-based sensing and signal processing by providing a comprehensive overview of the research performed previously as well as highlighting conceptual advantages, current progress and future challenges in the field.

## 1. Introduction

Neuromorphic computing has recently garnered much interest. This emerging technology promises lower power and lower latency than established methods for sensing and computing by emulating principles of information processing in the brain. A key concept of neuromorphic computing is event-based sensing, inspired by the property of sensory neurons in the nervous system to preferably respond to changes of the sensed quantity, rather than to continuously report its current level. The latter approach is represented by the established principle of periodic sampling, alongside the signal processing toolbox based on Discrete Fourier Transform (DFT). While the periodic sampling/DFT approach has been extremely successful, it suffers from several inherent weaknesses. First, it is in practice restricted to bandlimited signals due to the aliasing problem. Second, periodic sampling may waste energy when signals change only intermittently. Third, there is an inherent limitation of the minimum achievable latency imposed by the sampling interval; when using DFT for signal processing this gets worse as it requires a window of samples.

Event-based sensing represents a family of sampling schemes where the signal drives the sampling. A sensing element, such as a pixel, a filter bank element, or a gas sensor, emits an event when the signal crosses a threshold. This sampling scheme is often called “send-on-delta.” Other, largely synonymous terms are “event-driven sampling,” “absolute-deadband sampling,” “Lebesgue-sampling,” among others; the specifics of event triggering allow for tuning of the algorithm (Vasyutynskyy and Kabitzsch, 2010). Previous work has analysed the suitability of signal-driven sampling schemes for different kinds of signals (Liu et al., 2019), highlighting that the send-on-delta sampling scheme is particularly suitable for signals that are sparse, intermittent, and not bandlimited.

The event-driven sensing paradigm has been pioneered in vision, but recently also found its way into other sensory modalities. Here, we provide a survey on event-based sensing and signal processing approaches in modalities: vision, sound, and olfaction. The purpose of this survey is designed to put these three modalities in context and provide an overview of the publications in the field.

Event-based vision sensors have evolved during the last decade from an existence almost exclusively limited to a few select research labs to now being widely available as commercial products from several manufacturers worldwide, with several established application domains. Their bio-inspired operation differs from traditional vision systems in that their sampling regime is not frame-based and periodic; instead, sampling at each individual pixel is driven by the signal itself. Just as Ganglion cells in the retina emit a spike when a certain feature (i.e., brightness) in their receptive field changes, event-based vision sensors emit an event when a pixel detects that brightness crosses a threshold (“send-on-delta”). Vision signals are often very broad-band and can require extremely short sensor latencies to be captured accurately, while also being sparse in time. These are favorable conditions for event-based sampling schemes (Liu et al., 2019).

The transition from periodic sampling to event-driven sampling has also been implemented in the auditory domain (Lyon and Mead, 1988). Again, close inspiration from biological auditory systems has guided system development, mimicking the operation of biological cochlea, the mechanical properties of which implement a filter bank that enables spectral decomposition of the audio signal, subsequently transduced by hair cells into spikes. The output of an event-based silicon cochlea is a sparse stream of digital address-events, that represent the address of active channels, with each channel representating a frequency band. In theory, silicon cochleas could increase the sensitivity to rapid changes in the spectral composition of audio signals, since they do not require windowed Fourier transform which inherently introduces a lag in signal processing. However, the bandlimited nature of auditory signals facilitates the choice of a periodic sampling frequency that will allow efficient processing within the expected variation of the signal (Liu et al., 2019). Nevertheless, substantial amounts of research have explored the principles of operation and demonstrated use cases for event-based silicon cochlea, which we summarize below.

As a third example of event-based sensing we cover Olfaction, the sense of smell. There is a long history of seeking bioinspiration for electronic nose systems. One perhaps representative example is the NEUROCHEM project that ran from 2008 to 2011 (Marco et al., 2013). It brought together scientists from different disciplines around bio-inspired olfaction systems. Olfactory stimuli are carried by turbulent dispersal, which imposes wide-band fluctuations on their concentration at a given point in space. The physical properties of turbulent processes indicate that odour dispersion results in an intermittent signal where long periods of “blanks” are interspersed with brief and wide-band “whiffs” (Celani et al., 2014). These turbulence-induced fluctuations can be very rapid, and carry information that could be helpful in locating odour sources—an essential task for foraging or mate-seeking biological agents as well as in robotic gas sensing, e.g., for environmental and factory monitoring, or disaster management (Mylne and Mason, 1991; Schmuker et al., 2016). Given that rapid fluctuations olfactory signals carry useful information, it is not surprising that progress in Olfactory Neuroscience has recently uncovered that animals can decode very short transients in olfactory stimuli (Szyszka et al., 2014; Erskine et al., 2019). In summary, olfaction signals could be very well amenable to event-based sensing since they combine sparseness and intermittent detection with very rapid fluctuations. However, the olfactory domain has also seen the least exploration from the three modalities that we cover in this survey, highlighting the great potential for future research.

### 1.1. Principles of Event-Based Signal Processing

Once an event is generated by detecting a threshold-crossing in the signal, it is emitted as a data structure typically containing two pieces of information: 1. An *address*, e.g., the coordinates of the pixel that emitted the event, or the index of the filter bank, or the gas sensor instance, and 2. the *time* of event creation. In real-time systems, time can represent itself and only the address of the sensor needs to be transmitted. This protocol is commonly called Address-Event Representation (AER).

Algorithms for AER signal processing are largely independent of periodic sampling, and therefore do not suffer from aliasing. Moreover, information acquisition is driven by the spatio-temporal changes in the signal itself, therefore inherently capturing the dynamics of the underlying scene, unlike frame-based systems where these dynamics first have to be reconstructed from the sequence of samples. AER algorithms also have promising properties for parallelization and composition. AER processing modules have the inherent capability to be assembled into hierarchical structures (Serrano-Gotarredona et al., 2009). This is due to the fact that the communication between the AER modules can be made completely asynchronous, i.e., without having to rely on central synchronization. Previous work has developed “glue modules,” such as AER splitters, mergers and mappers that connect these individual processors together (Gomez-Rodriguez et al., 2006).

Despite all these benefits, conventional signal processing algorithms cannot be used for these systems. Unlocking the full potential of AER systems often requires designing new algorithms, starting from first principles in the event-based paradigm. The review of existing event-based algorithms is therefore an integral part of this survey. Notably, there exists a research community around event-based signal processing and control (Miskowicz, 2016), but so far the cross-pollination to brain-inspired event-based sensing has been limited.

### 1.2. Structure of This Paper

In this paper, we survey the literature published on neuromorphic and event-based sensing in vision, auditory and olfactory sensing systems. Our goal was 2-fold: to identify shared challenges for event-based sensing in these modalities, and to provide a collection of references covering relevant work in these three areas to facilitate research bridging between areas.

Event-based vision is by far the most advanced modality concerning event-based technology and applications, and therefore it takes up most of the space in the survey section of this paper. Auditory event-based sensing has received much less attention, and olfactory even more so. Comprehensive review and survey papers covering these modalities are much harder to find than for vision, and we hope that our contribution will help the inclined reader to identify relevant primary research in these areas.

Finally, the focus on sensory systems indicates that there is a gap in research in the area of more general event-based *processing*. We discuss this at the end of the paper and point out some domains that may show potential for event-based approaches to data analytics.

The rest of this paper is organized as follows: in section 2 we review the existing surveys on event-based signal processing approaches. Section 3 reviews primary literature on event-based vision systems. Event-based auditory systems are covered in section 4, and olfactory systems in section 5. Section 6 provides a summary of the references covered. Finally, in section 7 we conclude the paper by discussing the main takeaways from this survey and potential future work in this area.

Readers may want to initially focus on sections 1 and 7 sections and treat the survey sections 2 to 5 as reference collections that may enable a “deep-dive” into each field.

## 2. Previous Surveys and Benchmarks

Among the first papers that review a relevant field is a survey on neuromorphic vision sensors (Etienne-Cummings and Van der Spiegel, 1996). Performed more than two decades ago, when the field was still at its infancy, that survey provides a history of implementing neuromorphic sensors. More recently a short survey of silicon retinas and cochleae has been presented (Delbruck and Liu, 2012), providing a history of recent advances in the area. In Delbruck (2016), a perspective on developments of event-based vision sensors, algorithms and applications over the period of 2002–2016 is presented. The most recent and likely most comprehensive survey on event-based vision contains “everything that has ever been written” on the topic (Delbrück, personal communication; Gallego et al., 2020).

Specialising on the design of VLSI neuromorphic circuits for event-based signal processing, in Indiveri (2008) an overview of selective attention systems based on neuromorphic winner-take-all networks, ranging from single-chip vision sensors to multi-chip systems is presented. In another work, a very short survey of neuromorphic chips is presented in Liu and Wang (2009), which introduces the required hardware and summarizes the applications.

A good discussion on recent developments in neuromorphic optical sensing and artificial vision is presented in Posch et al. (2014). The paper introduces the functionality of biological retinas and then provides an overview of existing neuromorphic vision systems and their implementation. Then the paper expands to a discussion on the design of silicon retinas and neuromorphic vision devices.

A mini-review of current neuromorphic approaches for vision, auditory and olfactory sensors (Vanarse et al., 2016) provides a useful review on some state-of-the-art approaches, but it covers only a small proportion of research in this area.

A literature survey and taxonomy of artificial olfactory systems is presented in Kowadlo and Russell (2008). In Moraud and Chicca (2011), a short review on the field of bio-inspired autonomous navigation and neuromorphic chemical sensing is presented. In Chicca et al. (2013), a brief review of neuromorphic olfactory sensors can be found. A review on neuromorphic approaches for artificial olfactory systems is performed in Vanarse et al. (2017).

To support continuous improvement of algorithms and methods, there is a need for challenging event-based datasets. Benchmark data sets exist that are specifically crafted to assist model design, refinement and testing using event-based signal processing algorithms. In one of the first major efforts, in Tan et al. (2015) some guidelines for the creation of neuromorphic vision benchmarks and challenges is presented. In Gibson et al. (2014a), a data set of 11 moving scenes recorded by a dynamic vision sensor is generated. In Li et al. (2017), using a dynamic vision sensor, 10,000 frame-based images are converted into event streams. The conversion is performed by repeated closed-loop smooth movement of frame-based images. In Serrano-Gotarredona and Linares-Barranco (2015), two sets of data are proposed released for event-based object recognition. One set was obtained by browsing poker card decks and the other was generated by displaying 10,000 moving symbols. In another work (Hu et al., 2016) mostly dynamic vision tasks like action recognition or tracking are targeted for benchmark data collection. In Zhu et al. (2018), a data set with a synchronized stereo pair event-based camera system for 3D perception is presented, which is collected in a variety of illuminations, environments and camera mountings. A data set is presented in Binas et al. (2017) which is a recording of DVS cameras in driving applications. The data are collected under different conditions like daytime, night, dry, wet surface, and different driving speeds. Several event-based algorithms and a remarkable JAVA framework for the DVS can be found at jAER (2021).

## 3. Event-Based Vision Systems

Machine vision has seen the greatest uptake of event-based sensing and signal processing approaches so far. There are many approaches to develop silicon retinas, examples of which include (Etienne-Cummings et al., 2000; Costas-Santos et al., 2007; Delbruck, 2008; Lichtsteiner et al., 2008; Delbrück et al., 2010; Matolin et al., 2010; Sulzbachner and Kogler, 2010; Camunas-Mesa et al., 2011, 2012; Leñero-Bardallo et al., 2011; Posch et al., 2011; Serrano-Gotarredona et al., 2013; Darwish et al., 2015; Garćıa et al., 2016; Zheng et al., 2016).

Event-based vision has clear advantages over frame-based approaches. First, event-based vision systems report the exact times of relative brightness changes rather than providing a snapshot of absolute brightness at all locations in the visual field. Absolute pixel illumination is not an invariant property of the environment (Lowe, 2004), and it has been hypothesized that this is the reason why many current algorithms fail in uncontrolled conditions (Reinhard et al., 2010).

Second, periodic sampling imposes restrictions on the amount of information that can be extracted from a scene. For example, it has been shown that the human eye can resolve visual dynamics at up to 1 KHz, because this is where natural scenes contain most of the information; Even a sampling rate of 60 Hz can lead to an information loss of around 75% (Akolkar et al., 2015a).

Third, event based sensors can achieve very short latencies that are only constrained by the minimal response time of the sensor, because they only collect data when something is happening, whereas frame-based systems are forced to obey the fixed inter-frame interval. Moreover, periodic sampling suffers from a trade-off between low latency and high inter-frame redundancy, whereas event-driven sampling schemes avoid transmitting temporally redundant information while maintaining the capability of low-latency response to changes. Low latency and avoidance of redundant information acquisition also largely eliminates motion blur.

These properties make event-based vision highly suitable for applications like robotic platforms (Mueggler et al., 2014), where standard cameras with their relatively high latency and computationally expensive sensing and processing pipeline are sub-optimal. It should however be noted though that low-power and low-bandwidth sensing can only be achieved in scenes with sparse activity. Richly textured visual scenes or auditory scenes with high levels of white noise might cause very high event rates, and in consequence power consumption.

### 3.1. Applications

Since proposed, these devices have found their way in many applications. Here we provide a review on applications of event-based vision systems.

#### 3.1.1. Tracking

Arguably, due to the nature of these sensors, tracking is the most straight forward application of DVS cameras. Tracking with conventional machine vision algorithms is a computationally expensive task. However, as DVS cameras only transmit changes in the images, they are inherently suitable for tracking moving objects. For this reason, among all the applications, the largest number of research is performed in tracking.

##### 3.1.1.1. Object Tracking

A hierarchical neuromorphic system for tracking objects is presented in Gómez-Rodŕıguez et al. (2011), where two processing layers work in cascade for first detecting moving objects and then tracking them with crossing trajectories. In Zong et al. (2018), MLS surface fitting and local plane fitting methods are employed to identify the images collected by a DVS camera for tracking objects. The system is tested on uniform and high speed motion and it is shown that it can filter noise and reach high accuracy and robustness.

Frame-based tracking systems become less accurate as the speed of objects increases. They are also susceptible to changes in illumination. The authors in Saner et al. (2014) approach this problem by combining a DVS and a frame-based camera, such that tracking is performed based on the frame-based system, but the DVS device is used to capture the information about the changes in the scene in the time interval between consecutive frames.

In Delbruck et al. (2015), a human vs. computer slot car racing is devised, where a DVS camera is used to track both cars and control the break and throttle of the racing car. The low latency provided by the DVS camera results in consistent outperformance of human drivers by the computer.

##### 3.1.1.2. Satellite Tracking and Space-Situational Awareness

The high dynamic range of an event-based camera is exploited to track satellites using a ground-based telescope in full daylight (Cohen et al., 2019). A dataset is also provided (Afshar et al., 2020).

##### 3.1.1.3. Multiple Object Tracking

Some works targeted specifically multiple object tracking, for example, Gómez-Rodŕıguez et al. (2010) that presents a cascade architecture for that purpose. In Linares-Barranco et al. (2015), a lattice structured FPGA framework has been presented that allows uncorrelated-event noise removal for tracking multiple objects. The system is capable of adapting itself to fast or slow and large or small objects.

##### 3.1.1.4. Stereo Tracking

Most tracking algorithms use one DVS camera which provides a 2D representation of the environment. Some works have tried to employ two cameras so they can build a 3D map of the environment resulting in a better tracking system, for example, Schraml et al. (2010b), that aimed at tracking people in 3D. The system is capable of detecting and tracking people within a 4 m range with a refresh rate of the depth map of up to 200 Hz. In another work (Müller and Conradt, 2012), two cameras independently track an object. Then a self-adjusted neural network maps the 2D angular coordinates into a Cartesian 3D position of the object.

##### 3.1.1.5. Camera Movement

Tracking can be used to calculate the camera movement. In Kim et al. (2008) a DVS is used to track accurate camera rotation to build a persistent mosaic of a scene. Another work (Reinbacher et al., 2017) proposed panoramic camera tracking. The authors show that the spatial position of the events is enough for simultaneous tracking and mapping, and there is no need for the appearance of the imaged scene point.

##### 3.1.1.6. Camera Pose Estimation

Using a probabilistic generative event model in a Bayesian filtering framework, a camera pose estimation algorithm is designed in Gallego et al. (2015, 2016). This research design the likelihood function used in the filter to process the observed events. Based on the physical characteristics of the DVS, the authors propose the use of the contrast residual as a measure of how well the estimated pose explains the observed events. The authors extend their work (Gallego et al., 2018a) by tackling the problem of accurate, low-latency tracking of a camera from an existing photometric depth map built upon classic dense reconstruction pipelines. Using cubic splines, in Mueggler et al. (2015c) the pose of a DVS camera is estimated by a smooth curve in the space of rigid-body motion, with the trajectory curve being optimized according to the incoming events.

##### 3.1.1.7. Feature Tracking

In some tasks, the camera tries to track some features in the scene. In Lagorce et al. (2015b), a DVS camera is used for tracking of multiple visual features. In the research, various kernels, such as Gaussian, Gabor, combinations of Gabor functions and arbitrary user-defined kernels are used to track features from incoming events. The trackers are capable of handling variations in position, scale and orientation by using multiple pools of trackers. In Ni et al. (2015) a pattern tracking algorithm is proposed, in which the pattern tracking iteratively updates the model location and orientation to match the 2D image plane based on the arrival of events. Since the algorithm tracks patterns, it is capable of tracking objects even if they are subject to transformations. Another example of using DVS cameras for tracking corner-event features is Alzugaray and Chli (2018a).

##### 3.1.1.8. Micro Particle Tracking

In Ni et al. (2012), an asynchronous event-based Hough circle transform is developed to track micro particles. The system allows for a robust multiobject position detection at a frequency of several kHz at a low computational cost. Measurements in turbulent fluid flows often require high-speed imaging techniques. These systems are usually limited by the amount of memory available on-board. In Drazen et al. (2011) a DVS camera is used for particle tracking which enables a 100-fold reduction in bandwidth and data storage. A fast-flow visualization method is presented in for tracking buoyant soap bubbles. The data analysis in this work relies on Kalman filters to associate the events with traces and to reconstruct the path and velocity of particles.

##### 3.1.1.9. Sub-atomic Particle Tracking

An extensive parallel tracking system is designed in Neri et al. (2015), that allows real-time tracking withe a latency of <1 μs. The retina architecture is organized in three main blocks. The first block is a buffer that stores the hit information according to a hold logic. This module gets activated when downstream modules are busy. The second block is a pool of engines that process the hits. And the third block calculates the track parameters. The authors present the testbeam results in Neri et al. (2017).

##### 3.1.1.10. Car Tracking

An embedded vision system for tracking cars has been designed in Litzenberger et al. (2006c) which offers a one millisecond timestamp resolution.

##### 3.1.1.11. Person Tracking

In Pikatkowska et al. (2012), the problem of multiple person tracking in the occurrence of high occlusions is addressed. The authors apply Gaussian Mixture Models for detection, description and tracking individuals.

##### 3.1.1.12. Robotics

In many robotic applications, the agility of robots is limited by their sensing pipeline. A DVS camera is used in Censi et al. (2013) for robot pose tracking to increase robot agility, demonstrating that tracking performance is unaffected by fast motion. An autonomous target tracking approach is proposed in Jiang et al. (2017) for a snake-like robot. Using the Hough transform based on spiking neural networks the target pole is detected as two parallel lines from the visual input. The pose and periodic motion features of the robot are combined to develop an adaptive tracking based on the estimated depth information. In order to design a tracker which is robust to temporal variations due to the relative movement at different velocity of camera and target, a new algorithm is developed in Glover and Bartolozzi (2017). The authors develop a particle filter that follows the target position within the spatio-temporal data, while rejecting the clutter events that occur as a robot moves in an environment. The tracker is used in a robot vision system.

#### 3.1.2. Classification

One main application of event-based cameras is in classification. Among the first studies that used an event-based camera for classification is Schraml et al. (2010a), in which an algorithm for pedestrian classification is proposed that makes use of density and distance metrics for clustering asynchronous events generated by scene dynamics. In Chen et al. (2012) an algorithm is developed for categorization of human postures that employs a combination of event-based hardware and bio-inspired software architecture.

In O'Connor et al. (2013), a method based on the Siegert approximation is proposed for integrate-and-fire neurons to map an offline-trained deep belief network onto an event-driven spiking neural network. They use this system in character recognition in presence of distraction. The event-based classification algorithms' performance lags far behind their frame-based counterparts. The authors in Sironi et al. (2018) cite two reasons, first the lack of low level representations and architectures, and second the lack of real-world event-based data-sets. To tackle these, the authors introduce an event-based feature representation and release a dataset for object classification.

#### 3.1.3. Stereo Matching

A variety of computer vision applications require a 3D structure of the real-world scene. This task is usually performed by a stereo vision, which consists of two cameras observing the same scene from two different angles. Since these two cameras capture slightly different pictures, an algorithm is needed to match corresponding pixels that are projections of the same scene in the images. In frame-based approaches, the frames from two digital cameras are processed pixel by pixel and the patterns that match in both stereo frames are found. When using event-based cameras, it is the events that should be processed to yield such information. This means that whole new set of algorithms are needed to perform this task. In this part of the paper, we review the works that are performed in this area.

In Dominguez-Morales et al. (2011), the existing frame-based stereo matching algorithms are discussed and then an AER stereo matching algorithm is proposed that exploits some of the principles in frame-based stereo matching. In Kogler et al. (2010, 2011a,b); Kogler (2016), the time difference between the received pixels is used as matching criterion. The authors use a global optimization scheme that is designed to deal with sparse data to minimize the matching cost. The work also designs a filter that analyzes the disparities around pixels. In Carneiro et al. (2013), a novel N-ocular 3D reconstruction algorithm is proposed that allows preserving the original dynamics of the scene. This results in a more robust 3D reconstruction.

In a research (Rogister et al., 2012), it is shown that matching on the timing of the events provides information about the 3D objects, when combined with geometric constraints using the distance to the epipolar lines. The approach is capable of filtering out the incorrect matches and can accurately reconstruct the depth model. Because of the geometry of the sensors, estimating the epipolar geometry constraints is difficult. In Benosman et al. (2011), it is shown that these constraints are a consequence of the static frames, and using event-based cameras can, to some extent, overcome this limitation. The authors present a model for asynchronous event-based vision that is used to derive a new concept of epipolar geometry based on the temporal information of the pixels.

##### 3.1.3.1. Cooperative Neural Networks

A modification of the cooperative network is used in Piatkowska et al. (2013, 2014) to store the history of the recent activity in the scene. This is used to serve as spatiotemporal context used in disparity calculation for the events. In this system, the network constantly evolves as events arrive, the network constantly evolves. The work then is further improved (Piatkowska et al., 2017) to reduce the error by over 50%. A dynamic cooperative neural network is used in Firouzi and Conradt (2016) in which the interaction between cooperative cells applies cross-disparity uniqueness-constraints and within-disparity continuity-constraints, to asynchronously extract disparity for each new event. This work is then extended in Dikov et al. (2017), where a spiking neural network is implemented on SpiNNaker.

A different approach is presented in Osswald et al. (2017) that unifies the domains of perceptual neuroscience and machine vision. In this research, a spiking neural network is proposed that is inspired by cooperative network of Marr and Poggio (1976) and is capable of computing stereo correspondence from the visual stream of neuromorphic vision sensors. Because of the dynamic properties of the neuromorphic neural networks, their co-localization of memory and computation and their size, these networks offer possible solution to the Von Neumann bottleneck problem, which is a promising platform for stereo vision systems.

##### 3.1.3.2. Gabor Filter

The use of Gabor filter in extracting information about the orientation of the object edges that produce the events is studied in Camuñas-Mesa et al. (2014a) and Camunas-Mesa et al. (2014). The authors apply the matching algorithm to the events produced by the Gabor filter instead of the events produced by the DVS, therefore increasing the number of constraints applied to the matching algorithm. Their results show that this technique improves the final 3D reconstruction.

##### 3.1.3.3. Using Single Camera

In conventional stereo matching algorithms, a set of camera from different angles are used to find a dense 3D structure of the scene. In Rebecq et al. (2016, 2018), however, it is investigated how one single DVS camera can be used to build a semi-dense 3D structure. DVS cameras have two characteristics that make this possible: they respond to edges, which naturally provide semi-dense geometric information about the scene and they provide continuous measurements of the scene. In another work (Kim et al., 2016), a single DVS 3D reconstruction algorithm is proposed which is based on three decoupled probabilistic filters, each estimating 6-DoF camera motion, scene log intensity gradient and scene inverse depth relative to a keyframe.

##### 3.1.3.4. Similarity Measure

Performing stereo matching requires a type of similarity measure that defines a criteria based on which the corresponding pixels are found. In Schraml et al. (2015) a cost function is proposed which uses a similarity measure based on event distributions. A novel feature descriptor is proposed in Zou et al. (2016) which can describe the local context or distribution of the event data and constructs an effective similarity measure for data matching. Considering the correlation of incoming events, in Eibensteiner et al. (2017), in addition to the spatial information, the time of the occurrence of the events is also used as part of the similarity measure. In Zihao Zhu et al. (2018), the velocity of the camera and a range of disparities are used to synchronize the positions of the events as if they were captured at a single point in time. The authors propose a novel cost over these time synchronized event disparity volumes that rewards similarity between volumes and penalizes blurriness. In Zhou et al. (2018), of the optimization of an energy function is designed to exploit small-baseline spatiotemporal consistency of events triggered across the image planes. To reduce the uncertainty of the estimation, a probabilistic depth-fusion strategy is developed. The method does not require the motion of the camera or prior knowledge about the scene.

##### 3.1.3.5. Verification Approaches

Stereo matching with DVS cameras is a new field and there is an emerging community of scientists that develop algorithms and methods for the problem. The existing ground truth data and evaluation platforms that are proposed for frame-based systems cannot be used for event-based systems. Therefore, there is a need for new metric and verification methods to measure the performance of the proposed algorithms. In Sulzbachner et al. (2010), a tool for synthetic scene generation, ground truth generation and algorithm verification is proposed. In another work (Kogler et al., 2013), a new approach for the evaluation of stereo matching algorithms is presented.

#### 3.1.4. Recognition

Object recognition is one of the main fields in machine vision and as a new technology, event-based cameras have found their way in the field. Camera sensor networks are a network of camera in an environment than collectively capture and process visual information. Due to the number of cameras, these systems require high computational power. In Teixeira et al. (2006), a pattern recognition algorithm is designed for a network of event-based cameras to identify some hand gesture signs. In Ahn et al. (2011), a bare hand gesture recognition algorithm is proposed that recognizes three gestures in rock-paper-scissors game. In Amir et al. (2017), an event-based camera and an event-based processor with one million spiking neurons are used for human gesture recognition. They report that their algorithm recognizes gestures with a latency of 105 ms. A hardware implementation of event-based data processing is presented in Hofstätter et al. (2011), where an event-based camera is used for object recognition.

Solving texture recognition task with an event-based sensor is targeted in Pérez-Carrasco et al. (2010), where the authors show that the recognition rate has not degraded when new sensors are used. In Negri et al. (2018), an event-based camera is used to recognize the shape of poker signs. Combining an event-based sensor with a convolutional neural network, an object recognition and orientation estimation algorithm is proposed in Ghosh et al. (2014), which shows very high accuracy at real-time speed. In Orchard et al. (2015), a spiking hierarchical model is presented for object recognition which show that the temporal information of the events can be used in object recognition in a simpler way than traditional methods.

An event-based camera is used in Reverter Valeiras et al. (2016) to solve the 3D pose estimation problem. While in frame-bases systems the sampling frequency is 30–60 Hz, the authors take advantage of event-based cameras and design a pose estimation algorithm that achieve a temporal resolution of resolution of several hundreds of kHz on a conventional laptop.

#### 3.1.5. Detection

Published reports of event-based cameras being used for detection are still comparably scarce. A face detection algorithm is proposed in Barua et al. (2016), in which a patch-based model for the events is developed. The designed system is capable of reconstructing 2,000 frames per second. In Cannici et al. (2018), two neural network architectures are proposed for object detection, where one network integrates events into surfaces and one that uses convolutional and max pooling layers to exploit the sparsity of camera events. An FPGA implementation of retinal ganglion cell model is designed in Moeys et al. (2016b) which detects moving objects. The authors use this processing in conjunctions with a DVS to extrapolate information about object position. Using a DVS, a car detection algorithm is proposed in Chen (2018) which by employing convolutional neural network handles motion blur and poor illumination conditions problems.

Hand gesture recognition is also studied in Lee et al. (2014), where a neuromorphic post-processing hardware is used. In this work, the motion trajectories of hands are detected, segmented and translated into discrete feature vectors. These feature vectors are then classified via hidden Markov models. In Alzugaray and Chli (2018b), an event-based camera is used for corner detection and tracking. They report promising results at with a speed four times higher than conventional algorithms. Corner detection is also studied in Clady et al. (2015), where a luminance-free method is developed.

Using event-based cameras, a line detection algorithm is proposed in Seifozzakerini et al. (2016, 2017), where Hough Transform is employed in spiking neural networks. In another work (Brändli et al., 2016), a line segment detector is proposed which tries to infer which events are caused by the motion of the same spatial feature by parameterizing the event streams as a set of line segments.

In event-based processing in textured scenes, millions of events are generated per second that require great computational power. To tackle this problem, a research (Mueggler et al., 2017a) proposes a method to reduce the stream of event to a corner event stream. They design a corner detection algorithm that reduces the event rate by a factor of 20. The commonly used Harris corner detector is used in Vasco et al. (2016), where the frames are replaced by a stream events. The research test their method on a DVS camera mounted on a robot.

Sun sensors are navigational tools used in spacecrafts to detect the position of the Sun. In Farian et al. (2015), an event-based sensor is designed that is composed of two lines of pixels that perform in parallel and two optical slits aligned above the chip. The sensor is capable of directly detecting the position of the Sun and so no further processing is required.

#### 3.1.6. Localization and Odometry

Fast localization is crucial in many applications like driving and maneuvering, which traditional cameras can seldom provide. Due to their sampling speed, event-based cameras are very suitable for localization and odometry. Among the first efforts to use event-based cameras for localization is Weikersdorfer and Conradt (2012), which adopts a condensation particle filter tracker and demonstrates robust performance at low computational cost. Another work (Weikersdorfer et al., 2013) proposes a localization and mapping method that offers real-time performance on standard computing hardware. A fast localization algorithm is proposed in Yuan and Ramalingam (2016), in which a fast spatio-temporal binning scheme is developed to detect lines from events. A 3-D model of the world is then constructed which is used to estimate sensor pose. In Milford et al. (2015), an event-based camera is used for simultaneous localization and mapping.

In one of the main first attempts in event-based odometry, a novel event-based tracking approach based on image-to-model alignment is combined with a 3-D reconstruction algorithm in a parallel fashion (Rebecq et al., 2017b). The proposed system runs in real time and supports high dynamic range input with strong illumination changes.

Odometry is to measure the ego-motion of a camera, used, e.g., in robotics. Event-based cameras have great potential for Odometry as they can track fast movement accurately without blurring and quantization. However, new algorithms are required to exploit the sensor's characteristic. The first research that uses event-based cameras in odometry is Kueng et al. (2016) and Mueggler et al. (2017b), in which the features are detected in the grayscale frames and then tracked using stream of events. These features are then fed to an odometry algorithm. In Zhu et al. (2017b), an event-based odometry algorithm is proposed that is asynchronous and provides measurement updates at a rate proportional to the camera velocity.

In Horstschäfer (2016), using an accelerometer and a gyroscope, an a technique is presented for image and event stabilization of an event camera. The camera is then used for visual odometry of a robot. An odometry algorithm is proposed in Rebecq et al. (2017a) which tracks a set of features via overlapping spatio-temporal windows to construct motion event frames. The results presented in the work suggest that their algorithm outperforms state-of-the art conventional approaches with much lower computational expense. In Mueggler et al. (2018) an algorithm is proposed in which the camera trajectory is approximated by a smooth curve in the space of rigid-body motions using cubic splines, which reduces the number of variables in trajectory estimation problems.

#### 3.1.7. Motion Detection

Motion detection has many applications and is an important area in machine vision research. The first research that uses an event-based camera for motion detection is presented in Ruedi (1996), where a simple retina of 23 by 23 pixels is used. A new motion detection algorithm is proposed in Barranco et al. (2009), where by integrating temporal feature results, a new matching algorithm with high stability is obtained. A clustering method is proposed in Schraml and Belbachir (2010) which exploits the sparse spatio-temporal representation of events for detecting moving objects. In Abdul-Kreem and Neumann (2015), the spatio-temporal filtering scheme suggested by Adelson and Bergen (1985) is adopted to make it consistent with the event representation. Finding representative features for motion information is another field of research which is targeted in Sullivan and Lawson (2017), where conventional neural networks are used to extract features.

A unifying framework is presented in Gallego et al. (2018b), in which several computer vision problems are solved: motion, depth and optical flow estimation. By maximizing an objective function, the point trajectories on the image plane are found that are best aligned with the event data.

Bio-inspired systems for motion detection have incorporated mechanisms from the visual system into spiking networks to achieve motion detection (Ridwan and Cheng, 2017; Dalgaty et al., 2018).

Optical flow is the pattern of apparent motion of objects in a scene created by its motion. In Rueckauer and Delbruck (2016), nine optical flow event-based algorithms are compared. To perform the comparison, a dataset of two synthesized and three real samples is created. The authors have made the data sets and the source codes for the algorithms publicly available. Some studies use neuromorphic networks for processing the output of event-based sensors. In Giulioni et al. (2016), an architecture for robust optical flow extraction with an analog neuromorphic multi-chip system is proposed. The algorithm uses a feed-forward network of analog neurons, and the computation is supported by the time of spike emissions. The optical flow is extracted based on time lag in the activation of nearby retinal neurons.

Finding the optical flow using a DVS camera is performed in Benosman et al. (2014), where it is shown that the precise optical flow orientation and amplitude can be estimated with a local differential approach on the surface defined by coactive events. In Bardow et al. (2016) an algorithm is designed that simultaneously finds the optical flow and the brightness of the images. In this work, a cost function is defined and minimized that contains the asynchronous event data and the spatial and temporal regularization within a sliding window with time interval.

An optical flow algorithm called adaptive block-matching is proposed in Liu and Delbrück (2018) which uses time slices of accumulated events, that are adaptively rotated on the input events and optic flow results. The rotation is performed in such a way to ensure the generated slices have sufficient features for matching.

Another example of event-based motion detection include Barranco et al. (2015a), the algorithm in Liu and Delbruck (2017) which mimics motion estimation methods used in MPEG, and the method developed in Gallego and Scaramuzza (2017) for angular velocity estimation.

In Barranco et al. (2014), a comparison between conventional vision algorithms and event-based cameras is performed. The authors show that due to the nature of event-based cameras, motion detection is much easier with these sensors, and they can easily outperform computer vision methods in accuracy and speed.

Event-based cameras have been reported to be evaluated for motion detection applications. For example Litzenberger and Sabo (2012) asks if event-based cameras can be used for optical motion analysis in sports, with a positive result. In Mueggler et al. (2015a), two DVS cameras are used to estimate the trajectory of objects that are thrown at a quadrotor. The object's trajectory is estimated using an Extended Kalman Filter with a mixed state space.

#### 3.1.8. Transportation Systems

Machine vision algorithms are widely used in transportation systems. The requirement for low latency processing plays to the strengths of event-based algorithms. A vision system is described in Litzenberger et al. (2006a) for counting vehicles simultaneously on up to four lanes of a motorway. The authors report fast, low power and robust vehicle counting. In another study (Litzenberger et al., 2006b), a silicon retina is used for vehicle speed estimation that measures the velocity of vehicles on four lanes simultaneously, under variable lighting and atmospheric conditions. A system for real-time classification of vehicles into cars and trucks is described in Gritsch et al. (2008), which achieves an accuracy of over 90%. An application in a pre-crash warning system is proposed in Kogler et al. (2009), where a silicon retina-based stereo vision algorithm achieves a temporal resolution of 1ms, across various lighting conditions.

#### 3.1.9. Healthcare

In recent years, computer vision has found many applications in healthcare, and applications of event-based processing are emerging in this field. Among the first attempts is the work published in Fu et al. (2008a,b), where a vision system is designed to detect accidental falls in elderly home care applications. Compared to frame-based methods, the system reports a fall at ten times higher temporal resolution and shows 84% higher bandwidth efficiency as it transmits fall events. In Belbachir et al. (2012) a stereo matching algorithm is used on two DVS cameras to provide a 3D vision system for fall detection that achieves over 90% positive detections. The authors argue that one advantage of using DVS cameras is privacy as it does not record the true images of the scenes.

In Ghaderi et al. (2015), a wearable mobility device is designed to assist the blind with navigation and object avoidance. In this system, two DVS cameras are used to provide a 3D vision, which is converted via an individualized head-related translate function into a 3D output sound. This device is then improved in Everding et al. (2016).

In order to decrease the transmission delay of visual and non-visual medical records, DVS cameras and edge computing are employed in Chen et al. (2017) reducing the transmission delay by 89.15–86.88%. Optical recording of neural activity requires cameras capable of detecting small temporal contrast with sample rate of 1 kHz. Using CMOS sensors is very challenging as they require high data rates of up to 1 Gb/s. To overcome this, a DVS camera is used for the task in Taverni et al. (2017), that suggests long-term use of the sensor in neural recordings can be very beneficial.

Using a DVS camera, a system is designed in Gaspar et al. (2016) which can be used as a retinal prosthesis or vision augmentation. An algorithm based on integrate and fire neuron model is used in this work to emulate temporal contrast sensitive retinal ganglion cells.

#### 3.1.10. Industry

Many industrial applications require very high sampling rate. For example, monitoring a turbine with thousands of rpm poses a serious challenge to frame-based vision systems. In Perez-Peña et al. (2011), a DVS-based surveillance video system is designed for ultra fast industrial environments, that monitors a machine with a rotating part at 6,000 rpm, with good results.

Flow visualization in wind tunnel testing is of crucial importance for practical applications. In Borer (2014), DVS cameras are used for tracking neutrally buoyant soap bubbles. The authors use three cameras to build a 3D reconstruction, where two cameras provide 3D vision and the third camera increases the reliability of detection in areas with poor lighting, poor background contrast or with reflections.

#### 3.1.11. Segmentation

Segmentation is the process of partitioning an image into multiple sets of pixels and is a common task in computational vision. In the first attempt to design a segmentation algorithm for event-based cameras, a contour detection algorithm is proposed in Barranco et al. (2015b), where structured random forests are used to find the location of contours and their border ownership. These contours are used for the segmentation of the scene. In Surovich et al. (2017) a dynamic segmentation of moving objects is proposed that uses a DVS with a linear polarizing filter. The authors use wavelet transform to analyze the local spatio-temporal content of the images. Segmentation requires high computational power, and in most applications, performing real-time segmentation is very difficult. In Thakur et al. (2017), the random walker algorithm is adapted to a spiking neuromorphic processor to perform real-time segmentation of the scene. The system can perform segmentation at the speed of 1,000 images per second.

Segmentation can benefit from color cues. Yet, the original DVS camera does not transmit color information. In Marcireau et al. (2018), a dichroic beam splitter is thus used to decompose the input light into red, green and blue lights, and then send them to three DVS cameras. The output of these cameras are then processed to perform color segmentation and tracking.

A new event-based protocol is proposed in Darwish et al. (2017), that suppresses spatial redundancies of event-based cameras. The activity of the event-based camera is limited to the effective and relevant information in the scene; therefore, the data flow is drastically reduced. The authors propose a cost-free image segmentation using their method.

#### 3.1.12. Robotics

Many tasks in robotics require reliable and low-latency sensing, hence posing a promising field for applying event-based cameras (Camuñas-Mesa et al., 2014b).

##### 3.1.12.1. Obstacle Avoidance

Among the first studies that used event-based cameras in a real robot is Clady et al. (2014), where these sensors are used to design a fast obstacle avoidance method. The use of event-based cameras in obstacle avoidance problem in robots was then continued in Blum et al. (2017) and Milde et al. (2017), where the authors show how it is possible to achieve functional robot obstacle avoidance control strategies using a mixed signal analog/digital neuromorphic processor with an event-based sensor.

In Milde et al. (2015), an obstacle avoidance system is designed which is based on optic flow. To extract optic flow, the authors use a plane fitting algorithm that estimates the relative velocity in a small spatio-temporal cuboid. The depth structure is then derived from the translational optic flow.

##### 3.1.12.2. Balancing and Control

Conradt's pencil balancing robot was using two event-based vision sensors to sense deviations from the vertical with low latency, and was demode at numerous conferences in the era (Conradt et al., 2009a,b). Event-based sensors are also used in Mueller et al. (2015a,b) for feedback control of mobile robotic systems. The work is continued in Singh et al. (2016) to investigate the problem of quadratically stabilizing a continuous time linear time invariant system using event-based cameras.

##### 3.1.12.3. Flying Robots

Low-latency processing of visual information is crucial for flying robots as thy require fast reactions. A new event-based method to compute optic flow for miniaturized indoor flying robots has been demonstrated in Conradt (2015), that can be embedded on the robot due to its low power requirements and small form-factor. In another work (Orchard et al., 2009), event-based cameras are used for planetary landing tasks. In Hordijk et al. (2017), the “local plane fitting” algorithm is extended to obtain an improved and more computationally efficient optical flow estimation method. The developed algorithms are implemented in a constant divergence landing controller on a quadrotor.

##### 3.1.12.4. Actuators and Manipulation

Many robots require vision for manipulating the environment. In Linares-Barranco et al. (2007), an event-based camera is used for visual sensing, processing and actuating a robot that mimics human behavior. To reproduce human movements, a spike processing strategy is proposed in Perez-Peña et al. (2013) that uses a silicon retina to find the trajectory of human movement. In another work (Jimenez-Fernandez et al., 2009), the actuators of a robot are controlled, based on the input from a camera, to move the robot on a line on the floor.

Precise information about the position of objects and manipulators is crucial in object manipulation tasks where the grippers lack force sensing. To provide a haptic feedback, an artificial retina is used in Bolopion et al. (2012) that provides high update rate of the moving objects and a frame-based camera is devised to provide the position of the object. In Ni et al. (2012), an event-based iterative closet point algorithm is proposed to track a micro-gripper's position. The authors use both a DVS camera and a frame-based camera, where the temporal precision of the asynchronous silicon retina is used to provide a haptic feedback to assist users during manipulation tasks, and the frame-based camera is used to retrieve the position of the object.

When grasping objects, human fingers have very sensitive touch receptors that enable us to apply the precise pressure needed to grasp items. Too low pressure can lead to grasping failure and too much pressure may damage the object. In Rigi et al. (2018), event-based cameras are used to develop algorithms for detecting incipient slip, stress distribution and object vibration. They compare their results with a high speed 1,000 fps camera and show good performance with a very small (44.1 ms) latency.

##### 3.1.12.5. Maneuvering and Navigation

The agility of robots is limited by the latency of their perception. Therefore, event-based cameras can by useful to support high speed robot maneuvers. To achieve a faster vision, the first onboard perception system for 6-DOF localization during high-speed maneuvering of a quadrotor is presented in Mueggler et al. (2014). A DVS is used in Delbruck et al. (2014) to extract motion parallax cues relating to 3D scene structure in the a navigation task, with better performance than frame-based approaches. A guidance system inspired by honeybee vision was proposed in Serres et al. (2016). The simulated bee is equipped with a compound eye comprising 10 sensors, two optic flow regulators that update the control signals, and three event-based controllers.

##### 3.1.12.6. Vision and Attention

In Klein et al. (2015), two DVS cameras are mounted in a robot head to provide vision. The authors designed an image stitching algorithm to represent a scene larger than the field of view of each of the retinas. In another work (Moeys et al., 2016a), a DVS camera is used on a head of a predator robot that follows a prey robot. Robot goalies require very fast reaction time which is hard to achieve with frame based systems. In Delbruck and Lang (2013) and Delbruck and Lichtsteiner (2007) a fast self-calibrating robotic goalie is designed which offers low latency and CPU load. In another work (Becanovic et al., 2002), a neuromorphic analog VLSI sensor is combined with a digital omni-directional vision system. The system is used on a robot for locating a ball and directing the actuators for a goal keeper robot. In order to achieve a fast interaction with the environment, an attention system is developed for a humanoid robot in Rea et al. (2013). The authors report low-latency systems for the attention task.

### 3.2. Algorithms

There are some studies that propose new ways of processing event based vision signals. In this section we review papers that have come with new algorithms for DVS camera data.

#### 3.2.1. Mapping

Convolutional neural networks (LeCun et al., 1989) inherently operate on frame-based principles. For many large-scale systems, event-based processing modules are impractical. In Pérez-Carrasco et al. (2013), an intermediate solution is presented. First, a database of training frames is generated by binning, i.e., collecting events during fixed time intervals. Second, a frame-driven convolutional neural network is trained to perform object recognition. Third, the learned parameters of the frame-driven convolutional network are mapped to an event-driven convolutional network. Finally, the timing parameters of the event-driven network are fine-tuned to optimize the recognition task.

#### 3.2.2. Filtering

In signal processing, filtering refers to the prepossessing that is applied on the signals for feature detection and extractions. In image processing for example, it is performed to find features like corners, edges, and so on. In Ieng et al. (2014), a filtering methodology is proposed for event-based cameras. The authors propose asynchronous linear and non-linear filtering techniques. In Bidegaray-Fesquet (2015) the effect of noise and uncertainty on levels on the filtering of event data is investigated. The authors analyze the errors in terms of standard deviation of the normal distribution.

#### 3.2.3. Lifetime Estimation

An algorithm is proposed in Mueggler et al. (2015b) that estimates the life-time of events from DVS cameras. The estimation is performed based on its velocity on the image plane. The application of such an algorithm is the construction of sharp gradient images at any time instant.

#### 3.2.4. Classification

Conventional neural networks cannot be directly applied to the classification tasks for event-based data. In Li et al. (2018), it is shown how the deep representation learned with an originally optimized CNN is efficiently transferred to the event-based classification task. In this method, a spike-event coding is used and implemented based on the subthreshold dynamic of the leaky integrate-and-file model.

#### 3.2.5. Compression

By only sending changes in the intensity of pixels, DVS cameras inherently perform high speed video compression. In Brandli et al. (2014), a decompression algorithm is proposed that performs an online optimization of the event decoding in real time. The system exhibits an adaptive compression ratio that depending on the activity in the scene can reach up to 1,800 for stationary scenes.

In order to design a compression algorithm for event-based data, an analysis on the spike firing mechanism and the redundancies of spike data generated from DVS is performed in Bi et al. (2018). The authors then propose a cube-based coding framework comprising three strategies, namely macro-cube partitioning structure, address-prior mode and time-prior mode.

A new compression algorithm for still images is proposed in Doutsi et al. (2015) which uses event-based sampling. In this algorithm, a bio-inspired filter is applied to the image and then the retinal-filtered image is fed to a sampler. To reconstruct the original image, the spike train produced by the sampler is decoded.

#### 3.2.6. Prediction

A spiking neural network with learnable delays is used in Gibson et al. (2014b) to predict temporal sequences of the incoming events from a DVS camera. The system is capable of learning the temporal structure of space-time events, adapt to multiple scales and is able to predict future events in a video sequence. Using a DVS camera, a method is presented in Kaiser et al. (2018) to learn movements from visual predictions. The proposed method consists of two phases. First is learning a visual prediction model for a given movement and second is minimizing the visual prediction error.

#### 3.2.7. High-Speed Frame Capturing

Event cameras only transmit light intensity changes in the scene, so they lack information about all the pixels. A method is proposed in Liu et al. (2017b), to recover a scene, in which the foreground exhibits fast motion and background is static. Frames taken from a conventional camera are first matched to events taken from a DVS camera, then the high-speed events are used to generate the image sequences between consecutive frames. Motion blur in frame-based cameras refers to the apparent streaking of moving objects in a photograph that occurs when part of the image being recorded changes during the exposure. In Pan et al. (2018), the blur generation process is modeled by associating the event data to a latent image. The method is called event-based double integral model that reconstructs a high frame rate, sharp video from a single blurry frame and its event data.

#### 3.2.8. Spiking Neural Networks

Due to specific characteristics of event-driven signals, conventional machine learning techniques cannot be directly used for these signals. Therefore learning systems should be designed that are specifically suitable for these data. A new evolving neural network is developed in Dhoble et al. (2012) that utilizes both rank-order spike coding, also known as time to first spike, and temporal spike coding. The authors implement the system for a classification problem on event-based data from a DVS camera. A novel method for training an event-driven classifier within a spiking neural network system is proposed in Stromatias et al. (2017), which uses the activity provided by an arbitrary topology of prior network layers to build histograms and train the classifier in the frame domain. This way of building histograms captures the dynamics of spikes immediately before the classifier. The system is applied to data from a DVS camera.

#### 3.2.9. Data Transmission

Normally, brain-machine interfaces emphasize faithful transmission of the recorded signals. An alternative approach is taken in Corradi and Indiveri (2015) that proposes a neural recording system is proposed for compressing data. This event-based system applies signal processing and neural computation to extract relevant information from the large amount of collected raw data. It transmits only the low-bandwidth outcome of the processing to remote computation modules.

#### 3.2.10. Fusion

In order to process the output of event-based cameras more accurately, different networks including convolutional and recurrent neural networks are ensembled in Neil and Liu (2016) to jointly solve a recognition task. The authors show that the performance of the algorithm is higher than individual networks.

#### 3.2.11. Hybrid Methods

Event-based vision systems offer fast visual processing with low computational requirements. However, high level visual processing, like, e.g., object recognition, is still a challenge for these devices. Some studies try to accomplish both objectives by combining the advantages of both systems. However, active vision systems need real time *and* high-level processing at the same time. In Sonnleithner and Indiveri (2011a,b, 2012), dedicated VSLI hardware is designed that implements an event-based network of spiking neurons for real-time processing in combination with a conventional vision system. A low-resolution event-based system responds in real-time to moving objects and produces fast reactive motor outputs. A conventional high-resolution machine vision system performs object recognition task.

In Weikersdorfer et al. (2014), a DVS and a frame-based camera are combined to produce a sparse stream of depth-augmented 3D points. The authors state a smaller amount of generated data and a continuous representation of motions as advantages of this system.

In order to combine the strength of both type of sensors, a frame-based video sensor is used along with an event-based camera in Leow and Nikolic (2015). The system is applied to a variety of applications including video-compression, foveated imaging on the moving objects, object tracking and velocity estimation.

#### 3.2.12. Matching

In Moser (2015), a new approach for matching event sequences is proposed that is based on Hermann Weyl's discrepancy norm.

#### 3.2.13. Feature Extraction

Feature extraction plays an important role in many machine learning applications. The problem is to determine which features from the signal should be extracted for processing, and how. In frame-based computer vision, the features are often defined as a function of the luminance of the pixels within an image. Temporal information of the scene is often not present, e.g., because the source material contains only still frames, or it is of comparably low precision, due to an underlying assumption that 24 frames/s are enough for applications with only moderately fast-changing scenes. Event-based cameras enable extracting different features as they capture temporal information of the scene at high precision. Feature extraction from event-based signals and their application in higher-level computer vision was the subject of many studies, that we review in the following.

##### 3.2.13.1. Vehicle Detection

A spiking neural network is introduced in Bichler et al. (2011, 2012) to extract temporally correlated features from spike-based dynamic vision sensors. A spiking neural network is used in this work, in which the neurons become sensitive to patterns of pixels with correlated activation times. The authors employ a spike-timing-dependent plasticity scheme, where the synapses that do not contribute to spike activation are depressed. The system is developed for detecting cars passing a freeway.

##### 3.2.13.2. Gesture Recognition

In Ahn (2012), local and global feature extraction methods are employed. First the local extraction method uses segmentation to extract smaller number of features from a long sequence of raw gesture events. This is called local because it only considers neighboring events. The global extraction transforms the local features to construct higher level features. The authors use an evolutionary algorithm for feature selection step.

##### 3.2.13.3. Robot Vision

A new time oriented visual feature extraction method is presented in Lagorce et al. (2013), which is base on echo-state networks. The method is unsupervised and is suitable for high dynamic environments.

##### 3.2.13.4. Hardware Implementation

An FPGA design of an analog-to-feature converter is presented in del Campo et al. (2013), which learns a dictionary of features from an event-based signal using matching pursuit and Hebbian learning. The code is sparse and suitable for neuromorphic processors. In Hoseini and Linares-Barranco (2018), using FPGA, a digital circuit is proposed for extracting frequency of rotating objects in real time. This feature can be used, along with other features for recognizing objects with rotating parts. In Yousefzadeh et al. (2015), a 2D convolution event-based processing unit it proposed to extract features from an input event flow. The system is highly parallel and can benefit from FPGA arrays.

##### 3.2.13.5. Optical Flow

In Koeth et al. (2013), it is shown how motion features with spatio-temporal profile can be self-organized using correlations of precise spike intervals. The authors show that their framework forms topologic organization of features in a way similar to human brain.

A luminance-free feature extraction method is proposed in Clady et al. (2017) which performs by mapping the distribution of optical flow along the contours of the moving objects into a matrix. Using speed-tuned temporal kernels, the optical flow is integrated locally or globally in a speed direction coordinate frame-based grid. This ensures that the features equitably represent the distribution of the normal motion with respect to the moving edges.

Most feature tracking methods rely on building a model of events and then computing optical flow by assigning events to corresponding models. This, however, results in a lower quality optical flow and shorter flow tracks. In Zhu et al. (2017a), a soft data association modeled with probabilities is presented which is computed in an expectation maximization scheme. To enable longer tracks, the method also computes the affine deformation with respect to the initial point and use the resulting residual as a measure of persistence. Thus, in this method, varying temporal integration, different for each feature is achieved.

##### 3.2.13.6. Feature Extraction Algorithms

Convolution of Gabor filters over the image is a standard technique in conventional feature extraction. In Tsitiridis et al. (2015), a spiking neural network is used to exploit the temporal nature of the signals. In this method, a biologically inspired Gabor feature approach is presented. The neural network has a hierarchical structure and provides a flexible approach that reduces computation. In Lagorce et al. (2015a), a new computational architecture for learning and encoding spatio-temporal features is presented, based on a set of predictive recurrent reservoir networks, competing via winner-take-all selection. The features in this method are learned in an unsupervised scheme.

In Chandrapala and Shi (2016), a novel architecture called the event-based Generative Adaptive Subspace Self-Organizing Map, for feature extraction is proposed. The system is inspired by cortical models of visual processing and is based on the concepts of sparsity and temporal slowness. In this model, layers of units can be cascaded to learn feature extractors with different levels of complexity.

In Peng et al. (2017), a feature extraction method is proposed which is based on bag of events probability theory. In this approach, each object is represented as a joint probability distribution of events. The authors claim five main advantages: First, the algorithm uses statistical learning methods with good mathematical foundations. Second, it has only one hyper-parameter, therefore reducing the effort spent in parameter tuning. Third, it is an online learning algorithm and does not require data collection. Fourth, it offers competitive results in real time. And finally, the approach requires very simple operations of addition and multiplication.

A new feature is proposed in Negri (2017) that is computed based on an extended local binary pattern (LBP) operator. The feature characterizes the connectivity of the asynchronous events in a two dimensional space. This feature can also be organized on histograms and combined with other features as histograms of oriented events.

A new set of features called time-surfaces is presented in Lagorce et al. (2017), which can be used to create a hierarchical pattern recognition architecture. In this model, the subsequent layers in the hierarchy extract increasingly abstract features using increasingly large spatio-temporal windows. The idea in this work is to use temporal information to create contexts in the form of time-surfaces which represent the temporal activity in a local neighborhood. The first layer in this hierarchy operates on a group of pixels and each subsequent layer feature unit performs operation on the output of previous feature unit.

##### 3.2.13.7. Hybrid Cameras

In order to combine the advantages of event-based cameras with frame-based technology, DAVIS cameras are proposed that consist of a frame-based camera and a DVS camera that fills the information gap between consecutive frames. In Tedaldi et al. (2016), a new feature extraction method is proposed for these type of cameras, in which the frames are first processed and features are detected. These features are then tracked in the blind time between the frames using the events. The system uses an iterative geometric a registration approach for feature tracking.

### 3.3. Analysis and Modeling

Analyzing and modeling the behavior artificial retinas can help understand, and hence devise ways to improve, their performance In this section we perform an overview on this line of research.

#### 3.3.1. Analysis

In Yousefzadeh et al. (2018) a study is performed on saccades, and it is shown that performing more saccades in different directions can result in more accurate object recognition. Since adding more saccades adds latency and power consumption, the authors propose an intelligent saccadic movement paradigm that reduces the number necessary saccades without sacrificing recognition accuracy. The authors then use a neural-network algorithm that learns to control the saccades, thus further reducing the latency.

The impact of fixational eye movements for a DVS camera is investigated in Löhr and Neumann (2018). The authors use a mirror system to generate the virtual eye movements, and analyze the shape of the Fourier spectrum of random motions of the recordings for stationary and moving features.

A DVS and jAER are integrated in Franco et al. (2013) and an analysis is performed on the system to describe a method to develop new applications in jAER. The paper also describes two applications of the system: tracking objects and constructing images from spikes.

In event-based systems, sampling is induced by the signal, rather than by an external clock. Therefore, mathematical theory of frame-bases systems cannot accurately be applied to these systems. In Grybos (2015), event-based signal processing and the application of irregular sampling theory and frames are studied for event-based signal reconstruction. The method consists of the application of the frame algorithm enhanced with adaptive weight method for signal reconstruction.

#### 3.3.2. Modeling

##### 3.3.2.1. Modeling Retinal Ganglion Cells

An event-based system is developed in Katz et al. (2012a) which models the behavior of retinal ganglion cells. A DVS camera sends the events to a micro-controller processing unit which implements an interrupt driven model of an Approach Sensitive Retinal Ganglion Cells (AS-RGC). Accurate modeling of retinal information processing is studied in Lorach et al. (2012), where the spatial and temporal properties of the ganglion cells in mammalian retina are modeled. A DVS camera is combined in this work with a model pulling non-linear sub-units to reproduce the parallel filtering and temporal coding that occurs in retina.

It is often assumed that neuromorphic technology, i.e., technology that mimicks biological neuronal computing architectures, can potentially help to understand the functionality of nervous system. However, existing neuromorphic systems usually fail to represent true behavior biological sensors and neurons. To overcome this, a neuroid-based ganglion retina cell model is presented in Argüello et al. (2013) that is capable of reproducing the essential features of the photo-receptor response to illumination. A real-time visual system emulator is developed in Kawasetsu et al. (2014) as a combination of hardware retina emulator and SpiNNaker chips, to model neural activity in the retina and visual cortex.

Modeling the early detection of approaching dark objects, which is the functionality of one type of retinal ganglion cells is studied in Liu et al. (2017a). The Java software and FPGA hardware implementation of this type of cells is conducted and it is shown that this model can have applications in developing attention systems.

##### 3.3.2.2. Modeling Event-Based Sensors

In Katz et al. (2012b), a high frame-rate USB camera is used to model the behavior of a DVS camera. The PS3-Eye camera performs at 125 fps, and is integrated into a jAER (2021) software which does real-time event-based sensor processing. A variational model is presented in Munda et al. (2018) that accurately models the behavior of DVS cameras, that is formulated on per-event-basis, where information about the asynchronous nature of events are incorporated via an event manifold induced by the relative time-stamps of events. This model enables the reconstruction of intensity images with arbitrary frame rate in real-time.

##### 3.3.2.3. Modeling of Cortical Mechanisms

In Tschechne et al. (2014), a new approach is proposed for modeling of cortical mechanism of motion detection. The model combines filters with spatio-temporal and direction specificity. The model is then used to record test stimuli, articulated motion and ego-motion.

### 3.4. Hardware Design

Several efforts to develop hardware systems dedicated to processing event-based vision signals exist. A hardware model of a selective attention mechanism implemented on a VLSI chip is presented in Indiveri (2000), that is used with analog neuromorphic circuits. The device can be used as a transceiver module for multichip neuromorphic vision systems. In Serrano-Gotarredona et al. (2006), a neuromorphic cortical-layer microchip is presented that computes processes 2-D convolutions of event-based vision data. The microchip is able to process 128 × 128 pixels and can be tiled up for higher resolutions. In another work (Vogelstein et al., 2007), a mixed-signal VLSI system is devised for spike-based vision processing. The model exploits arbitrary and re-configurable connectivity between cells in the multichip architecture.

A new vision hardware system called CAVIAR is developed in Serrano-Gotarredona et al. (2009), in order to propose computational neuroscience and machine vision that allows construction of modular, multilayered, hierarchical and salable sensory processing learning and actuating systems. The system is a massively parallel hardware that consists of a retina, programmable kernel, WTA chip, spatio-temporal processing chip, AER mapping and splitting FPGA and a computer-AER interfacing FPGA.

In Bartolozzi et al. (2011) a robotic vision system is proposed that comprises two DVS cameras with a dedicated processor, a General Address Event Processor and a FPGA that connects the sensors to the processors. A software module collects the events for further processing. The system is capable of interaction with real world in real time.

The HMAX model was proposed (Serre et al., 2007) to truly model the visual cortex (Riesenhuber and Poggio, 1999, 2000a,b). An event-based implementation of the model is proposed in Folowosele et al. (2011) to show its ability in classifying basic shapes.

## 4. Event-Based Auditory Systems

Digital audio recording devices decompose signals using classical digital signal processing techniques. In biological auditory systems, sound signals are decomposed into frequency bands by the mechanical properties of the basilar membrane in the cochlea. Hair cells transduce the band-passed components into neural pulses that are then propagated to higher auditory areas in the brain. Auditory information like speech, music, and environmental noise is temporally structured. The brain is thought to achieve a computational advantage by exploiting the timing of action potentials to code information, compared to mere rate codes. Several studies have thus explored the potential of event-based processing in auditory processing, which we review in this section.

The development of silicon cochleas for signal processing purposes has seen significant effort. Interested readers are referred to Chan et al. (2007), Wen and Boahen (2009), Liu et al. (2010), Koickal et al. (2011), Wang et al. (2015), Yang (2015), and Jiménez-Fernández et al. (2017).

### 4.1. Applications

#### 4.1.1. Localization

Using two microphones and a pair of silicon cochlea, a neuromorphic sound localization system is proposed in van Schaik et al. (2009) and Yue-Sek Chan et al. (2010). The algorithm proposed in this work is adaptive and supports online learning. A binaural event-based sound localization is presented in Finger and Liu (2011), which implements a spike-based correlation of the spikes and measures the Inter-aural Time Differences (ITD) between the arrival of a sound to the two cochleas. When a spike arrives, the algorithm updates a possible distribution of ITD, which offers a faster solution to the problem compared to conventional cross-correlation methods. A probabilistic model for sound localization using silicon cochlea is presented in Anumula et al. (2018). Instead of using the timing of the spikes to find ITDs, this work uses spikes to support a distribution model of the ITDs.

In order to enhance the perceptual sensation on a hearing aid system, a neuromorphic sound localization circuit is designed in Park et al. (2013). The system is comprised of leaky integrate-and-fire neurons that are optimized to reduce the synaptic circuit noises.

#### 4.1.2. Echolocation

A bat-inspired, event-based localization method is proposed in Abdalla and Horiuchi (2008), which produces qualitatively similar, direction-dependent, spectral features in the same ultrasonic frequency range used by the big brown bat. The input sound signal generated by the an ultrasonic cochlea are sent to spiking neurons which then convert these spikes to spike trains. The authors use pattern recognition algorithms to estimate the azimuth and elevation of the ultrasonic chirps.

#### 4.1.3. Micro-Doppler Sonar

The relative velocity of objects to an observer can be estimated via the frequency shift of the sound produced by the objects. This phenomenon is used by some animals like bats and dolphins to navigate and locate objects. A system for micro-Doppler sonar is presented in Figliolia et al. (2015), which uses a silicon cochlea with acoustic fovea and AER.

#### 4.1.4. Speech Recognition

A speech perception algorithm is proposed in Näger et al. (2002) which uses a model of human cochlea with spiking neural network. The network employees synaptic plasticity to learn patterns by establishing characteristic delay structures.

The authors of Jansen and Niyogi (2009) design point process models to operate on sparse detector-based representation of speech signals and apply them to speech recognition tasks. They show that this system can operate at a comparable level to a basic hidden Markov model.

A speaker-independent isolated digit recognition system is designed in Abdollahi and Liu (2011), that works based on cochlear image maps based on the spikes from a silicon cochlea. The cochlear maps were found by means of time-binned spike counts, low-pass filtered spike trains and the Radon spike count method. These maps are then fed to a support vector machine for classification.

A spiking neural network composed of three types of integrate-and-fire neurons is proposed in Miró-Amarante et al. (2017) that is capable of recognizing vowel phonemes. The neural network is described in VHDL for detecting Spanish words.

#### 4.1.5. Speaker Identification

A method for speaker identification employing a silicon cochlea and limit-cycle statistics is proposed in Chakrabartty and Liu (2010). The authors employ a Gini-support vector machine classifier and use spike rates, inter-speak-interval distributions and inter-spike-velocity features. In Li et al. (2012), auditory features representing fading histograms of inter-spike intervals and channel activity distributions are extracted from the output of a silicon cochlea. Then a linear support vector machine is used to classify the feature vectors.

#### 4.1.6. Sound Recognition

In Jäckel et al. (2010), a sound recognition system is designed that uses a silicon cochlea and classical hidden Markov model. The system is trained to recognize two different sound of a clap or a bass drum in presence of noise. A neuromorphic auditory system for feature extraction and an artificial neural network are used in Cerezuela-Escudero et al. (2016) to recognize 12 musical notes in presence of white noise.

#### 4.1.7. Sensor Fusion

In Chan et al. (2012), a pair of silicon cochlea and a silicon retina are combined on a robotic platform to allow the robot to learn sound localization through visual feedback and a sound localization algorithm. This work is an extension on the work presented in van Schaik et al. (2009) and Yue-Sek Chan et al. (2010), where only silicon cochlea was used for localization. The authors report that the combination with a silicon retina improves sound localization.

The combination of visual and auditory information can help resolve ambiguities in sensing. In Akolkar et al. (2015b), event-based vision and auditory systems are combined to design a collision detection algorithm for application in robotics. Collisions are distinguishable from mere occlusions by on the sound the collision produces. Salient sensory events must therefore be detected by vision and auditory systems at the same time. This requires very high temporal resolution and is challenging for frame-based systems.

In Rios-Navarro et al. (2015), an event-based camera and auditory systems are used together to measure the rotation frequency of a motor. The system uses a FPGA and performs in real-time.

#### 4.1.8. Feature Extraction

In Anumula et al. (2018), the effectiveness of frame-based features generated using spike counts and constant event binning is investigated. The authors propose a pre-processing method which applies an exponential kernel on the events to better preserve timing information. In Acharya et al. (2018) the authors extend their feature extraction approach to fixed number of bins and fixed bin size methods.

## 5. Event-Based Olfactory Systems

Olfaction plays an essential role in many activities, e.g., food foraging, trail following, mating, bonding, navigation, and detection of threats. Artificial olfaction has great potential in many areas, including hazard detection, food safety, industrial and environmental monitoring, disaster management, crop monitoring, medical diagnosis, among others. However, gas sensor technology still lags far behind what is available the visual and auditory domains. Designing artificial olfactory systems faces a number of challenges, including coping with slow sensor recovery, sensor drift compensation, concentration-invariant recognition, orthogonalization of odor patterns, mixture separation, and odor identification against complex backgrounds and interferents, among others (see Pearce et al., 2003 for a review). Event-based approaches could help mitigate some of these issues.

The front end of the olfactory system in vertebrates consists of a large number of olfactory receptor neurons that fire action potentials upon encountering volatile chemicals in inhaled air. Humans have around 5 million olfactory receptor cells, each of which expresses a only one of about 350 possible olfactory receptors. Different olfactory receptors differ in their molecular receptive ranges. One type of receptor may respond to a range of odorants, and one odorant typically elicits responses in a range of receptor. A combinatorial code emerges that encodes the identity of an odorant by the pattern of olfactory receptors that is activated (Bieri et al., 2004).

Artificial olfactory systems follow a similar principle, where arrays of sensors with partly overlapping response characteristics are combined in a frame-based manner and subsequently processed to extract activation patterns that can be assigned to particular odorants. In practice, gas sensor signals are often collected over long periods of time, like tens of seconds to several minutes, and subsequently averaged to remove turbulence-induced signal variations and other noise. This approach invariably introduces latency and removes any information-containing turbulence-induced information from the signal. In contrast, event-based olfactory systems try to mimic the key principles of biological olfaction, by transmitting events only when the gas concentration changes.

Here, we review gas sensing approaches that use direct inspiration from biology, dedicated hardware systems for neuromorphic gas sensing, and algorithms that have been suggested to improve gas sensing using neuromorphic principles.

### 5.1. Bio-Inspired Olfaction Systems

#### 5.1.1. Vertebrate Olfactory System

An artificial chemosensing system is presented in White et al. (1998) and White and Kauer (1999) which is based on neural circuits of the vertebrate olfactory system. An array of chemosensors which are designed to produce similar response to olfactory sensory neurons is used as input that produces spatiotemporal patterns. These patterns are recognized by a delay line neural network. The system is devised to encode the vapor identity by the spatial patterning of activity in the neural network and vapor intensity is encoded by response latency. The identity and intensity information are then separated into two distinct codes. This serves as a discriminator among organic vapors.

Inspired by olfactory structure of mammals, an artificial olfactory bulb is presented in Jing et al. (2016), which consists of olfactory receptor neurons and mitral, granule, periglomerular and short axon cells. The model transforms the input of gas sensors into neuron spikes that simplifies the feature generation step. The system is used in liquor classification.

#### 5.1.2. Insect Olfactory System

Since the output of odor sensors is usually real-time, continuous, noisy, lacks a precise onset signal and accurate classification often requires temporal information, many neuronal network models fail to operate properly in practice. To investigate the potentials and suitability of biomimetic classifiers for real-world sensor data, a research is performed in Diamond et al. (2016). In this work, inspired by insect antennal lobe, a generic classifier is designed to identify 20 individual chemical odors.

In Pearce et al. (2013, 2014), a biologically-constrained neuromorphic spiking model of the insect antennal lobe is presented that detects the concentration of chemical components of a material. The system is dynamic and uses winner-takes-all or winnerless competition depending on the inhibition and symmetry of its connections. The authors employ spike timing-dependent plasticity in their model and show that this is able to organize weights into a stable configuration.

#### 5.1.3. Honeybee Olfactory System

The honeybee's olfactory pathway is decomposed into its local circuits and processing stages in Hausler et al. (2011) and Schmuker et al. (2011). The authors demonstrate functional role of these organs and build a model a spiking neuronal network models of them by designing a probabilistic classifier. In another work (Kasap and Schmuker, 2013), also inspired by honeybee antennal lobe, unsupervised learning of the lateral inhibition structure is presented. The authors use inhibitory spike-timing dependent plasticity in a computational model for multivariate data processing. In this system, the inhibitory connectivity self-organizes to reflect the correlation between input channels. It is shown in this paper that local learning produces an inhibitory connectivity that reduces channel correlation and is suitable for a neuromorphic architecture. This line of work further gave rise to the first published implementation of a spiking network for multivariate pattern recognition on neuromorphic hardware (Schmuker et al., 2014).

#### 5.1.4. Stereo Olfaction

In Rochel et al. (2002), inspired by animal olfactory systems in tracking odors, a stereo sniffing system is designed that tracks specific odors. In this system, first the gas-concentration gradient is estimated, and then the gas is recognized. The authors use spiking neural networks to implement this biologically inspired system.

### 5.2. Hardware Systems

In this section we review the research that have developed hardware systems specifically designed for odor recognition.

#### 5.2.1. Hardware Design

A hardware architecture for chemical classifiers is presented in Abdel-Aty-Zohdy et al. (2010) which takes advantage of Sampling Spiking Neural Networks (SSNN). The chip records learning statistics and can be used in parallel with other SSNN co-processors to build very large systems.

#### 5.2.2. VLSI

Among the first attempts to develop a VLSI spiking neuromorphic is Koickalb et al. (2004) and Pearce et al. (2005). In this work, an olfactory bulb model, a reduced 70-element chemosensor array and the silicon implementation are presented. An adaptive neuromorphic VLSI olfaction device with on-chip chemosensor array is designed in Koickal et al. (2006, 2007). The system processes temporal spiking signals and classifies the odors. In Hsieh and Tang (2012) a VLSI neuromorphic spiking neural network olfactory system is designed that uses sub-threshold oscillation and onset-latency representation, in order to reduce power consumption. The authors use the synaptic weights between the mitral and cortical cells according to an spike-timing-dependent plasticity learning rule.

#### 5.2.3. Gas Recognition

In another work (Ng et al., 2011), a CMOS gas recognition chip is presented which encodes sensor outputs into spikes with the firing delay mapping the strength of the simulation. The circuit processes the spikes and looks for match within a library of spatio-temporal signatures. Exploiting fundamental characteristics of the olfactory pathway, a simple spike based gas recognition technique is presented in Al Yamani et al. (2012a,b). The system is designed for detecting ethanol, methane and carbon monoxide. Gas recognition is performed in this system by looking for a match within a library of spatio-temporal spike patterns. In Hassan et al. (2015), instead of the logarithmic time encoding model, spike codes are formed from transient features (similar to Muezzinoglu et al., 2009), thus eliminating the need for regression.

### 5.3. Modeling and Algorithms

Algorithms for modeling olfactory systems and for improving recognition performance have been proposed by a range of studies.

#### 5.3.1. Accelerated Event-Based Gas Sensing

In Drix and Schmuker (2021) a Kalman-filter based algorithms is described that can decode fast transients (in the order of one second) from metal-oxide sensors. It uses an event-based signal representation to detect gas onset with high temporal precision. An application in gas source direction detection is demonstrated.

#### 5.3.2. Event-Based Source-Distance Estimation

In Schmuker et al. (2016) and event-based (albeit non-spiking) algorithm is proposed that exploits turbulence-induced signal fluctuations to estimate the distance of a gas source in a wind tunnel.

#### 5.3.3. Neural Networks

A spiking olfactory bulb model is implemented in programmable logging and combined with a Hebbian learning rule in Guerrero-Rivera and Pearce (2007). The system is able to store attractors which correspond to odor patterns, and can classify learnt odors.

In Beyeler et al. (2010), the topology of biological networks is studied, and it is analysed how network activity depends on various parameters of the theoretical models. The authors' aim is to shed light on how network structure relates to filtering and enhancement of recognition performance.

#### 5.3.4. Neuromorphic Design

A network model of the glomerular layer of the mammalian olfactory bulb is implemented in neuromorphic hardware in Imam et al. (2012). In Martinelli et al. (2009), an artificial olfactory system is proposed and implemented on FPGA. The model is based on a direct spike conversion of the input signal and digital glomerular signal processing for spikes.

#### 5.3.5. Computational Modeling

Inspired by the biological principle of distributed coding, and olfactory receptor neurons converging in a chemotopic fashion onto glomerular units in the olfactory pathway, in Raman et al. (2006a), a computational model of chemical sensor arrays is presented. The work presents a monotonic concentration-response model that maps the sensor inputs into a distributed activation pattern across receptor models. Then a self-organizing model of chemotopic convergence is used to simulate the projection onto glomerular units in the olfactory bulb.

#### 5.3.6. Contrast Enhancement

In order to enhance the discrimination of multivariate patterns from gas sensor arrays, a signal processing model is presented in Raman et al. (2006b), which improves the separability between input odor patterns. The model captures chemotopic convergence of sensory neurons onto the olfactory bulb and center on-off surround lateral interactions. The features are projected onto a two dimensional lattice which results in odor-specific spatial patterning. These patterns are then fed to a network of mitral cells to enhance the contrast among odors and decouples odor identity from intensity.

#### 5.3.7. Spike Latency

To study the hypothesis that neurons transmit the most meaningful information via the first spikes, and that spike latency acts as a descriptor of the information content, an artificial sensory system is designed with a single layer of spiking neurons in Di Natale (2011). The authors assessed the system's capability to discriminate between distinct chemicals and mixtures, and studying the effect of lateral inhibition. The authors considered both the spikes latency and the average firing rate as the output of the network. Experiments with the system show that the average firing rate offers the best separation among stimuli, while latency offers discrimination in shorter time. These results aligned with observations in biological olfaction.

A latency-based e-nose system is designed in Chen et al. (2011) to achieve power-efficient, compact and robust gas identification, using rank order and spike distance classification algorithms.

## 6. Summary of Reviewed Research

In this paper we reviewed research on event-based signal processing, focusing on visual, auditory and olfactory systems. We did not attempt to cover event-based control systems, since the field is an independent area with large amount of research that would require a separate review. There is also a wide range of research on neuromorphic engineering which we did not cover in this paper due to constraints in scope and size; readers so inclined are referred to other excellent survey papers (Cauwenberghs, 1998; Liu and Wang, 2009; Liu et al., 2009; Nawrocki et al., 2016; Vanarse et al., 2016; James et al., 2017; Schuman et al., 2017).

**Figure 1:**
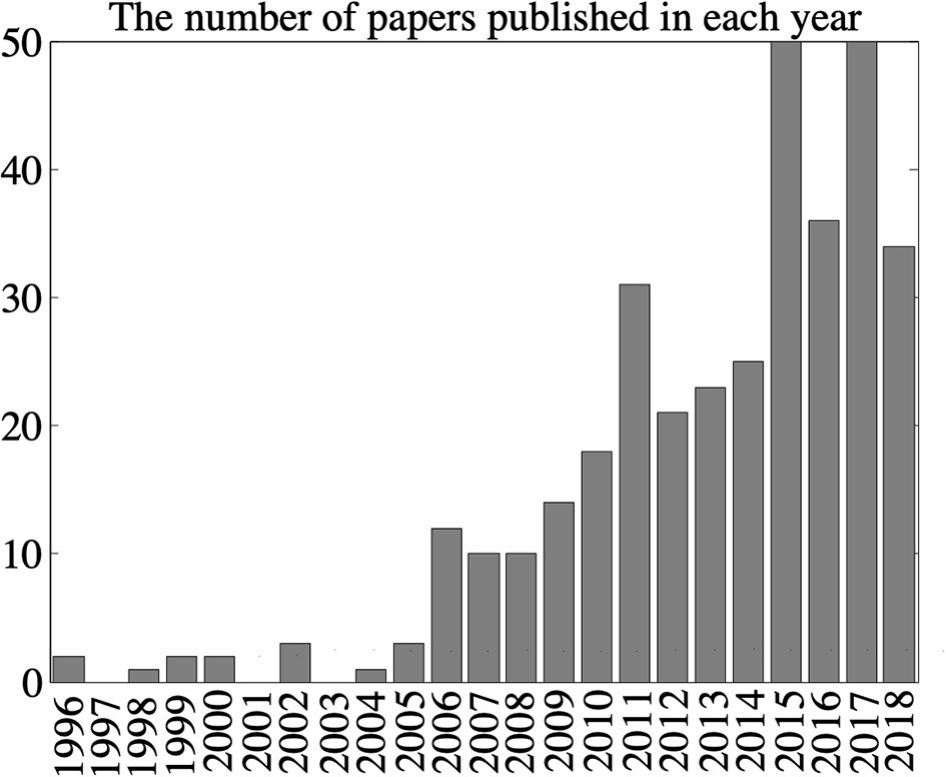
The number of reviewed papers on event-based visual, auditory and olfactory systems in each year since 1996.

There is an interesting trend in the volume of published research in event-based visual, auditory and olfactory systems during the last two decades (Figure 1). Few papers have been published prior to 2006, when a significant increase in interest saw a surge in publications. After 2006, there is steady increase in the research on the subject, indicating that the potential of the technology has been discovered.

Tables 1, 2 summarize the structure of this paper and the papers reviewed.

**Table 1:** The paper structure.

**Sensor type**	**Category**	**Subcategory**	**Papers reviewed**
Retina	Reviews, benchmarks	Previous reviews	Neuromorphic vision and cameras (Etienne-Cummings and Van der Spiegel, 1996; Delbruck and Liu, 2012; Posch et al., 2014; Gallego et al., 2020), Period of 2002–2016 (Delbruck, 2016), neuromorphic chips (Liu and Wang, 2009),
			VLSI neuromorphic circuits (Indiveri, 2008), spiking neural networks (Brette et al., 2007)
		Benchmarks	Guidelines for benchmark creation (Tan et al., 2015), dataset (Gibson et al., 2014a; Li et al., 2017), object recognition (Serrano-Gotarredona and Linares-Barranco, 2015),
			action recognition and tracking (Hu et al., 2016), 3D perception (Zhu et al., 2018), driving applications (Binas et al., 2017)
	Applications	Tracking	Object tracking (Gómez-Rodŕıguez et al., 2011; Saner et al., 2014; Delbruck et al., 2015; Zong et al., 2018), multiple object (Gómez-Rodŕıguez et al., 2010; Linares-Barranco et al., 2015), camera movement (Kim et al., 2008; Reinbacher et al., 2017),
			feature tracking (Lagorce et al., 2015b; Ni et al., 2015; Alzugaray and Chli, 2018a), stereo tracking (Schraml et al., 2010b; Müller and Conradt, 2012), camera pose (Gallego et al., 2015, 2016, 2018a; Mueggler et al., 2015c),
			micro-particle tracking (Drazen et al., 2011; Ni et al., 2012; Borer et al., 2017), subatomic particle tracking (Neri et al., 2015, 2017),
			car tracking (Litzenberger et al., 2006c), persons tracking (Pikatkowska et al., 2012), robotics (Censi et al., 2013; Glover and Bartolozzi, 2017; Jiang et al., 2017)
		Stereo matching	Stereo matching (Kogler et al., 2010, 2011a,b; Benosman et al., 2011; Dominguez-Morales et al., 2011; Rogister et al., 2012; Carneiro et al., 2013; Kogler, 2016), single camera (Kim et al., 2016; Rebecq et al., 2016, 2018),
			cooperative neural network (Piatkowska et al., 2013, 2014, 2017; Firouzi and Conradt, 2016; Dikov et al., 2017; Osswald et al., 2017), gabor filter (Camunas-Mesa et al., 2014; Camuñas-Mesa et al., 2014a),
			similarity measure (Schraml et al., 2015; Zou et al., 2016; Eibensteiner et al., 2017; Zhou et al., 2018; Zihao Zhu et al., 2018), verification approaches (Sulzbachner et al., 2010; Kogler et al., 2013)
		Classification	Pedestrian classification (Schraml et al., 2010a), human postures (Chen et al., 2012), character recognition (O'Connor et al., 2013),
			feature representation (Sironi et al., 2018)
		Detection	Object detection (Moeys et al., 2016b; Cannici et al., 2018), car detection (Chen, 2018), corner detection (Clady et al., 2015; Vasco et al., 2016; Mueggler et al., 2017a; Alzugaray and Chli, 2018b),
			line detection (Seifozzakerini et al., 2016, 2017), face detection (Barua et al., 2016), Sun detection (Farian et al., 2015)
		Localization	Localization (Weikersdorfer and Conradt, 2012; Yuan and Ramalingam, 2016), localization and mapping (Weikersdorfer et al., 2013; Milford et al., 2015)
		Odometry	Odometry (Horstschäfer, 2016; Kueng et al., 2016; Mueggler et al., 2017b, 2018; Rebecq et al., 2017a,b; Zhu et al., 2017b)
		Motion detection	Motion detection (Adelson and Bergen, 1985; Ruedi, 1996; Barranco et al., 2009, 2015a; Schraml and Belbachir, 2010; Abdul-Kreem and Neumann, 2015; Liu and Delbruck, 2017; Ridwan and Cheng, 2017; Sullivan and Lawson, 2017; Dalgaty et al., 2018; Gallego et al., 2018b),
			quadrotor (Mueggler et al., 2015a), comparison with machine vision (Barranco et al., 2014), motion detection in sport (Litzenberger and Sabo, 2012),
			velocity estimation (Gallego and Scaramuzza, 2017), optical flow (Benosman et al., 2014; Bardow et al., 2016; Giulioni et al., 2016; Rueckauer and Delbruck, 2016; Liu and Delbrück, 2018)
		Recognition	Texture recognition (Pérez-Carrasco et al., 2010), hand gesture recognition (Teixeira et al., 2006; Ahn et al., 2011), human gesture recognition (Amir et al., 2017),
			object recognition (Hofstätter et al., 2011; Ghosh et al., 2014; Orchard et al., 2015), shape recognition (Negri et al., 2018), pose estimation (Reverter Valeiras et al., 2016)
		Transportation	Counting vehicles (Litzenberger et al., 2006a), vehicle speed estimation (Litzenberger et al., 2006b), vehicle classification (Gritsch et al., 2008),
			pre-crash warning (Kogler et al., 2009)
		Healthcare	Fall detection (Fu et al., 2008a,b; Belbachir et al., 2012), medical data transmission (Chen et al., 2017),
			blind assistant (Ghaderi et al., 2015; Everding et al., 2016), neural activity recording (Taverni et al., 2017), prosthesis (Gaspar et al., 2016)
		Industry	Surveillance systems (Perez-Peña et al., 2011), wind tunnel (Borer, 2014), measuring rotation (Rios-Navarro et al., 2015)
	Robotics	Obstacle avoidance	Obstacle avoidance (Clady et al., 2014; Milde et al., 2015, 2017; Blum et al., 2017)
		Balancing and control	Balancing (Conradt et al., 2009a,b), feedback control (Mueller et al., 2015a,b; Singh et al., 2016)
		Flying robots	Computing optic flow (Conradt, 2015), landing (Orchard et al., 2009), planetary tasks (Hordijk et al., 2017)
		Actuators, manipulation	Mimicking human behavior (Linares-Barranco et al., 2007; Perez-Peña et al., 2013), line following (Jimenez-Fernandez et al., 2009), haptic feedback (Bolopion et al., 2012; Ni et al., 2012),
			grasping (Rigi et al., 2018)
		Maneuvering, navigation	Maneuvering (Mueggler et al., 2014), navigation (Delbruck et al., 2014; Serres et al., 2016)
		Vision and attention	Vision (Klein et al., 2015), predator robot (Moeys et al., 2016a), robot goalies (Becanovic et al., 2002; Delbruck and Lichtsteiner, 2007; Delbruck and Lang, 2013), humanoid robot (Rea et al., 2013)
	Algorithms	Algorithms	Mapping (Pérez-Carrasco et al., 2013), filtering (Ieng et al., 2014; Bidegaray-Fesquet, 2015), lifetime estimation (Mueggler et al., 2015b), classification (Li et al., 2018),
			compression (Brandli et al., 2014; Doutsi et al., 2015; Bi et al., 2018), prediction (Gibson et al., 2014b; Kaiser et al., 2018), high-speed frame capturing (Liu et al., 2017b; Pan et al., 2018),
			spiking neural networks (Dhoble et al., 2012; Stromatias et al., 2017), data transmission (Corradi and Indiveri, 2015), matching (Moser, 2015)
			hybrid methods (Sonnleithner and Indiveri, 2011a,b, 2012; Weikersdorfer et al., 2014; Leow and Nikolic, 2015), fusion (Akolkar et al., 2015b; Rios-Navarro et al., 2015; Neil and Liu, 2016)
		Feature extraction	Vehicle detection (Bichler et al., 2011, 2012), gesture recognition (Ahn, 2012), robot vision (Lagorce et al., 2013),
			hardware implementation (del Campo et al., 2013; Yousefzadeh et al., 2015; Hoseini and Linares-Barranco, 2018), optical flow (Koeth et al., 2013; Clady et al., 2017; Zhu et al., 2017a),
			feature extraction algorithms (Lagorce et al., 2015a; Lagorce et al., 2017; Tsitiridis et al., 2015; Chandrapala and Shi, 2016; Negri, 2017; Peng et al., 2017), hybrid cameras (Tedaldi et al., 2016)
	Analysis and modeling	Modeling	Retinal ganglion cells (Katz et al., 2012a; Lorach et al., 2012; Argüello et al., 2013; Kawasetsu et al., 2014; Liu et al., 2017a), event-based sensors (Katz et al., 2012b; Munda et al., 2018),
			cortical mechanism (Tschechne et al., 2014)
		Analysis	Saccades (Yousefzadeh et al., 2018), eye movements (Löhr and Neumann, 2018), jAER (Franco et al., 2013; jAER, 2021), reconstruction (Grybos, 2015)
	Hardware design	Hardware design	VLSI (Indiveri, 2000; Vogelstein et al., 2007), multichip neuromorphic (Serrano-Gotarredona et al., 2006), modular design (Serrano-Gotarredona et al., 2009), robotic vision (Bartolozzi et al., 2011),
			modeling visual cortex (Serre et al., 2007)

*The reviewed papers on event-based vision systems categorized based on applications and methodologies*.

**Table 2:** The paper structure.

**Sensor type**	**Category**	**Subcategory**	**Papers reviewed**
Cochlea	Reviews, benchmarks	Previous reviews	Neuromorphic cochlea (Vanarse et al., 2016)
		Benchmarks	No benchmark reported for silicon cochlea
	Applications	Localization	Online learning (van Schaik et al., 2009; Yue-Sek Chan et al., 2010), ITD (Finger and Liu, 2011), hearing aid system (Park et al., 2013), probabilistic model (Anumula et al., 2018)
		Eco location	Bat head (Abdalla and Horiuchi, 2008), Micro-Doppler Sonar (Figliolia et al., 2015)
		Speech recognition	Speech recognition (Näger et al., 2002; Jansen and Niyogi, 2009), digit recognition (Abdollahi and Liu, 2011), Spanish vowel (Miró-Amarante et al., 2017),
			speaker identification (Chakrabartty and Liu, 2010; Li et al., 2012)
		Sound recognition	Clap or a bass (Jäckel et al., 2010), musical notes (Cerezuela-Escudero et al., 2016)
		Sensor fusion	Localization (Chan et al., 2012), collision detection (Akolkar et al., 2015b), rotation frequency (Rios-Navarro et al., 2015)
		Feature extraction	Feature extraction (Acharya et al., 2018; Anumula et al., 2018)
Olfactory	Reviews, benchmarks	Previous reviews	Artificial olfactory systems (Kowadlo and Russell, 2008), neuromorphic odor tracking (Moraud and Chicca, 2011), neuromorphic olfactory sensors (Chicca et al., 2013)
			neuromorphic olfactory systems (Vanarse et al., 2017), biological receptors (Narusuye et al., 2003)
		Benchmarks	No benchmark reported for silicon olfactory
	Animal olfactory	Vertebrate olfactory	Vertebrate olfactory system (White et al., 1998; White and Kauer, 1999), mammals (Jing et al., 2016)
		Insect olfactory	Insect antennal lobe (Pearce et al., 2013, 2014; Diamond et al., 2016)
		Honeybee olfactory	Honeybee's olfactory pathway (Hausler et al., 2011; Schmuker et al., 2011), honeybee antennal lobe (Kasap and Schmuker, 2013)
		Stereo olfaction	Stereo olfaction (Rochel et al., 2002)
	Hardware systems	VLSI	VLSI spiking neuromorphic system (Koickalb et al., 2004; Pearce et al., 2005; Hsieh and Tang, 2012), adaptive neuromorphic VLSI olfaction (Koickal et al., 2006, 2007)
		Hardware classifier	Sampling spiking neural networks (Abdel-Aty-Zohdy et al., 2010), CMOS gas recognition chip (Ng et al., 2011), gas recognition (Al Yamani et al., 2012a,b),
			logarithmic time encoding model (Hassan et al., 2015)
	Modeling and algorithms	Event-based signal processing	Extracting information from turbulent processes (Schmuker et al., 2016; Drix and Schmuker, 2021)
		Neural networks	Spiking neural olfactory bulb (Guerrero-Rivera and Pearce, 2007), networks topology (Beyeler et al., 2010)
		Neuromorphic design	Glomerular layer (Imam et al., 2012), direct spike conversion (Martinelli et al., 2009)
		Computational modeling	Chemical sensor arrays (Raman et al., 2006a)
		Contrast enhancement	Contrast enhancement (Raman et al., 2006b)
		Spike latency	Spike latency analysis (Di Natale, 2011), spike latency (Chen et al., 2011)

*The reviewed papers on event-based auditory and olfactory systems categorized based on the type of sensors, applications and methodologies*.

## 7. Conclusion and Discussion

Event-based sensing and signal processing has been applied to many applications, with promising results and several conceptual advantages. First, event-based systems only collect meaningful information; therefore, the redundant data are not transferred and processed, enabling a more efficient encoding scheme. These systems can operate in an asynchronous fashion, not limited by the constraints induced by a global clock. Second, information is reported instantaneously, in contrast to periodic-sampling systems that quantize based on their sampling rate. Also, temporal information on very short timescales can be captured, without the constraints imposed by the Nyquist Theorem. Third, event-based sensors provide the potential for lower power operation when sampling sparse signals, which can be an advantage in power-constrained scenarios like mobile devices or medical implants. Fourth, many event-based systems are implemented in a modular fashion, allowing to increase computational power by composition and parallelism.

Nevertheless, event-based systems are still in their infancy, especially in the auditory and olfactory domain. This technology provides promising potential, but also comes with a set of unique challenges, outlined below.

The greatest bottleneck for growth in neuromorphic sensing is probably that a comprehensive theoretical framework to formally describe and analyse event-based sensing and signal processing algorithms has yet to emerge. This hampers the development of event-based algorithms and applications. Cross-pollination from engineering-focused event-based research communities may provide a way forward.

The lack of a theoretical framework also complicates the translation of traditional machine learning algorithms from the frame-based into the event-based domain. While attempts of such translations have been successful, they invariably resulted in a performance penalty compared to the frame-based implementation (although this penalty can sometimes be very small). The opposite direction is also a challenge: interfacing asynchronous, event-based systems with frame-based, clocked digital systems. A naive approach is to simply create frames from the event-based signal representation, but clearly this is not optimal since it risks to render the advantages of event-based sensing and signal processing void. Future research is therefore expected to focus on developing machine learning techniques that are specifically designed for event-based systems. A promising line of research could exploit the inherent compatibility of event-based sensing with spiking networks, potentially in combination with operation on accelerated neuromorphic hardware systems.

In addition to translation from frame-based algorithms into the event-based domain, Traditional machine vision and signal processing approaches already offer a great number of tools and techniques that help solve many common tasks, e.g., image enhancement, image restoration, depth identification, etc., which are readily available in widely-used and well-documented software packages. However, researchers building event-based approaches often must start from scratch. The barrier for adopting event-based technology for tasks outside basic research is therefore rather high, and sometimes the cost may be perceived too high to outweigh the gains. A standard toolbox for event-based signal processing could be a game-changing asset to boost the accessibility of this promising technology.

Most applications described for visual event-based signal processing are simple tasks of detection and tracking. Conventional machine vision algorithms are now developed for much more sophisticated tasks. Interesting and relevant tasks like face recognition, human behavior analysis, medical diagnosis, product inspection, etc. are currently far beyond what could reasonably be achieved with event-based vision. This is likely not due to an inherent limitation of the event-based approach; after all, the human brain can perform all these tasks and it operates in an event-driven fashion. Rather, improvements in neuromorphic hardware and, perhaps more importantly, event-driven algorithms will be needed to compete with state-of-the-art machine vision, speech recognition and gas sensing solutions.

The vast majority of event-based systems until now have been designed for vision, with applications in the auditory domain emerging, and prototypes having been demonstrated in olfaction. Other areas of data processing that do not explicitly deal with sensing a physical quantity are less well-explored in the event-based signal processing community, in spite their inherent suitability for the processing scheme; For example, areas like internet security, traffic data analysis, human behavior analysis, finance, physical experiments, healthcare data, social media, video surveillance, etc., generate data that can be interpreted as events. For example, the data in traffic systems comprise some events, like accidents, breaks, turns, etc., that could be processed in event-based scheme. In finance, events like an large cargo ship getting stuck and blocking a major trade route can affect the price of oil. In healthcare, changes in blood pressure, blood sugar, heart beat rate can indicate specific problems. Research in these areas has yet to be permeated by event-based data processing strategies.

Event-based sensing and signal processing also provides a few interesting avenues for research that may be crucial for the future development of the field; for example, exploration of event-based noise and noise-tolerance, “anti-patterns” for event-based sensing, signal compression, and cryptography.

While noise reduction in event-based vision has been addressed previously (Padala et al., 2018; Xie et al., 2018), there remains a need for a theoretical treatment for the problem. Also, the effect of the noise on existing algorithms should be studied. Future algorithms should be developed that are robust to such noise.

“Anti-patterns” for event-based signal processing refer to specific types of data or environment where event-based approaches struggle or fail, while traditional approaches cope well or have been engineered to overcome issues. For example, textured visual scenes can cause extremely high event-counts that could overload the event transmission fabric. This problem effectively limits the pixel count of event-based vision sensors as a function of bus capacity. Similar anti-patterns could exist in event-based olfaction and audio processing, but have yet to be identified, studied, and have solutions provided for. Often, a look at neuroscience could suggest promising solutions. For example, the mammalian retina already provides a fascinating wealth of signal processing before any spikes are generated (Baden et al., 2016).

Signal compression in the event-based domain is an interesting topic. Event-driven sampling of sparse signals inherently implements a compression of the input information compared to periodic and thus temporally redundant sampling. Still there is a need for more research on data compression for these sensors, especially in the visual domain where event counts even for sparse signals can grow very quickly when using high-resolution sensors. We reviewed some approaches in this direction, but more theoretical analysis and practical algorithms could help develop more efficient compression mechanisms.

Cryptography plays an important role when dealing with sensitive data. Many algorithms exist for efficient encryption of sensitive audio and video signals. This area of research is mostly absent from current trends in the event-based research community. Yet, event-based signals might be sensitive and thus require encryption before transmission. Specifically designed encryption algorithms for event-based data is an important domain which has not been targeted yet.

Finally, specific to olfaction, a large challenge is the availability of powerful sensors. Current gas sensing technology lags behind olfactory capabilities of animals, and even insects. A particular problem is temporal resolution, at least if portability and low power consumption are desired. Event-based approaches exist to narrow the gap between technology and biology (e.g., Drix and Schmuker, 2021). Improvements in gas sensing technology will without doubt catalyze progress in event-based olfaction as well.

## Author Contributions

Both authors listed have made a substantial, direct and intellectual contribution to the work, and approved it for publication.

## Conflict of Interest

The authors declare that the research was conducted in the absence of any commercial or financial relationships that could be construed as a potential conflict of interest.
